# Antibiotics-Induced Intestinal Immunomodulation Attenuates Experimental Autoimmune Neuritis (EAN)

**DOI:** 10.1007/s11481-024-10119-9

**Published:** 2024-05-31

**Authors:** Alina Sprenger-Svačina, Ines Klein, Martin K. R. Svačina, Ilja Bobylev, Felix Kohle, Christian Schneider, Finja Schweitzer, Nadin Piekarek, Mohammed Barham, Maria J. G. T. Vehreschild, Helmar C. Lehmann, Fedja Farowski

**Affiliations:** 1 Department of Neurology, Faculty of Medicine and University Hospital of Cologne, University of Cologne, Cologne, Germany; 2 Department of Neurology, St. Katharinen-Hospital, Frechen, Germany; 3 Department II of Anatomy, Faculty of Medicine and University Hospital of Cologne, University of Cologne, Cologne, Germany; 4 Experimental Medicine, Faculty of Medicine, University Hospital of Cologne, University of Cologne, Cologne, Germany; 5 Department I of Internal Medicine, Faculty of Medicine and University Hospital of Cologne, Cologne, Germany; 6 Department of Internal Medicine II, Infectious Diseases, Goethe University, University Hospital Frankfurt, Frankfurt Am Main, Germany; 7 German Centre for Infection Research (DZIF), partner site Bonn-Cologne, Brunswick, Germany; 8 Department of Neurology, Klinikum Leverkusen gGmbH, Leverkusen, Germany

**Keywords:** Autoimmunity, Guillain-Barré Syndrome (GBS), Immune neuropathies, Gut microbiota, Intestinal barrier

## Abstract

**Background:**

The composition of gut microbiota plays a pivotal role in priming the immune system and thus impacts autoimmune diseases. Data on the effects of gut bacteria eradication via systemic antibiotics on immune neuropathies are currently lacking. This study therefore assessed the effects of antibiotics-induced gut microbiota alterations on the severity of experimental autoimmune neuritis (EAN), a rat model of Guillain-Barré Syndrome (GBS). Myelin P0 peptide 180–199 (P0 180–199)-induced EAN severity was compared between adult Lewis rats (12 weeks old) that received drinking water with or without antibiotics (colistin, metronidazole, vancomycin) and healthy rats, beginning antibiotics treatment immediately after immunization (day 0), and continuing treatment for 14 consecutive days. Neuropathy severity was assessed via a modified clinical score, and then related to gut microbiota alterations observed after fecal 16S rRNA gene sequencing at baseline and after EAN induction. Effectors of gut mucosal and endoneurial immunity were assessed via immunostaining. EAN rats showed increased gut mucosal permeability alongside increased mucosal CD8^+^ T cells compared to healthy controls. Antibiotics treatment alleviated clinical EAN severity and reduced endoneurial T cell infiltration, decreased gut mucosal CD8^+^ T cells and increased gut bacteria that may be associated with anti-inflammatory mechanisms, like *Lactobacillus* or *Parasutterella*. Our findings point out a relation between gut mucosal immunity and the pathogenesis of EAN, and indicate that antibiotics-induced intestinal immunomodulation might be a therapeutic approach to alleviate autoimmunity in immune neuropathies. Further studies are warranted to evaluate the clinical transferability of these findings to patients with GBS.

**Graphical Abstract:**

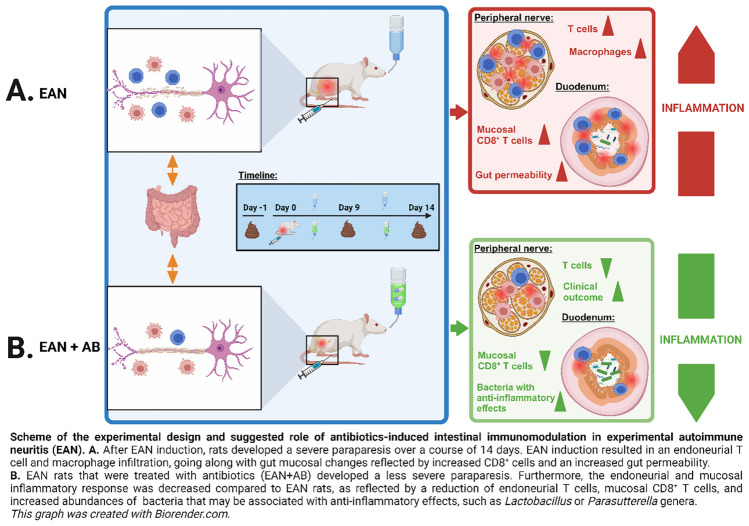

**Supplementary Information:**

The online version contains supplementary material available at 10.1007/s11481-024-10119-9.

## Introduction

Guillain-Barré Syndrome is an acute autoimmune disease that leads to inflammatory demyelination of peripheral nerves (Florian et al. 2021). It is considered the most common cause of acute flaccid paralysis with a global incidence of 0.6–4 per 100 000 individuals per year. Endoneurial accumulation of macrophages and peripheral nervous system (PNS) myelin antigen-reactive T cells are histopathological correlates of GBS, even though the exact histopathological features differ among the different subtypes (Kiefer et al. 2001; Súkeníková et al. 2024). Molecular mimicry mechanisms, driving this autoantibody-mediated immune reaction against peripheral nerve gangliosides and other, yet unknown neural epitopes, are considered to be the reason for a timely association of GBS with respiratory (Svačina et al. 2021) and especially gastrointestinal infections like *Campylobacter jejuni* enteritis (Shahrizaila et al. 2021).

Recent studies demonstrated a correlation between gut microbiota composition, pathogenic microorganisms like *Campylobacter jejuni,* and ganglioside autoantibody production in a murine GBS model (Brooks et al. 2017), and revealed that a treatment with beneficial gut bacteria ameliorates the course of experimental autoimmune neuritis (EAN), an established rat model for GBS that implies autoimmune T cells and macrophages leading to neuroinflammation and demyelination in the PNS (Shin et al. 2013). Furthermore, gut microbiota, that refer to all microorganisms found in the gut, play a crucial role in developing and maintaining intestinal homeostasis and immune tolerance (Shi et al. 2017). Unfavorable alterations of gut microbiota composition with the depletion of beneficial organisms as one of the main features have been found to also exacerbate other autoimmune diseases such as Multiple sclerosis (MS) or Crohn’s disease, by increasing gut mucosal permeability and altering antigen presentation to mucosal T cells (Kinashi and Hase 2021). Suitably, the induction of experimental autoimmune encephalomyelitis (EAE), an animal model of MS, led to an attenuated clinical course in germ-free mice lacking intestinal bacteria colonization (Lee et al. 2011). These germ-free mice also produced lower levels of intestinal pro-inflammatory cytokines and showed increased systemic regulatory T cell levels (Tregs) (Lee et al. 2011). These FOXP3^+^ Treg cells play a crucial role in preventing autoimmunity also in the PNS, since reduced blood Treg levels were shown to be predictors of EAN severity (Hörste et al. 2014).

We therefore hypothesized that an eradication of intestinal bacteria via antibiotics treatment might modulate intestinal and systemic cellular immune effectors and thus exert beneficial effects in EAN, which is a novel approach that has not yet been investigated in peripheral neuropathies.

There is a need for novel therapeutic approaches in GBS, since GBS is still fatal in about 5% of the cases, and about 14% of GBS patients are left with a severe disability despite the availability of established therapies like intravenous immunoglobulins or plasma exchange (Rajabally and Uncini 2012).

Therefore, this study lays the groundwork for future translational studies, and demonstrates that antibiotics-induced immunomodulation attenuates EAN.

## Methods

### Animals

Animal experiments were carried out according to the guidelines of local state authorities (Landesamt für Natur, Umwelt und Verbraucherschutz Nordrhein-Westfalen). Rats were single-housed in individually ventilated cages with sawdust bedding in pathogen-free conditions and a 12 h light/dark cycle, with water and standard food provided ad libitum. Seventeen (7 male/ 10 female) adult Lewis rats (aged 12 weeks; Janvier, France) were used for this study. EAN was induced in sex-matched cohorts (n = 5 rats per cohort) via a subcutaneous hind paw injection (under isoflurane anesthesia) of 100 µg of the neuritogenic myelin P0 peptide 180–199 (Zhu et al. 2001) (Genosphere Biotechnologies, France) in complete Freund´s adjuvant (Sigma-Aldrich, USA) containing heat-inactivated mycobacterium tuberculosis H37 Ra protein (2 mg/ml; BD, USA). Up from day 0, immediately after immunization with myelin P0 peptide 180–199, one cohort received drinking water enriched with colistin (2000 U/ml; Hikma Farmacêutica, Portugal), metronidazole (8 mg/ml; Braun, Austria) and vancomycin (0.1 mg/ml; Hikma Farmacêutica, Portugal), a second EAN cohort received antibiotics-free regular drinking water. Antibiotics intake was assured via daily control of the drinking volume, and subsequent daily renewal of the antibiotics-enriched drinking water. Seven age- and sex-matched Lewis rats served as controls and did not receive any intervention. All EAN rats were sacrificed at day 14 after EAN induction, control rats were sacrificed parallelly. A modified clinical score (Kieseier et al. 2000) was exerted to objectify the clinical course of EAN: 0—no impairment, 1—aberrance from normal behavior, 2—reduced tail tone, 3—absent righting, 4—gait ataxia, 5—mild paraparesis, 6—moderate paraparesis, 7—severe paraparesis, 8—tetraparesis, 9—moribund, 10—death.

### Stool sample collection

Six pieces of solid feces per animal were collected prior to EAN induction as well at day 9 and 14 after thereafter, and parallelly in healthy control rats. Fecal samples were immediately stored at -80 °C for further analysis.

### Genomic desoxyribonucleic acid (DNA) extraction

Bacterial genomic DNA (gDNA) was extracted from frozen naïve fecal samples via the FastDNA™ SPIN Kit for Soil (MP Biomedicals, Solon, OH, USA) to be able to amplify the 16S ribosomal ribonucleic acid (rRNA) gene from the gDNA in a second step, according to the manufacturers’ instructions.

### Bacterial 16S rRNA gene amplicon sequencing

16S rRNA gene amplicon sequencing was performed on the Illumina MiSeq platform (Illumina, San Diego, CA, USA) as outlined in the Illumina 16S Sample Preparation Guide (Illumina 2016). Briefly, the V3-V4 region of the bacterial 16S rRNA gene was amplified from 12.5 ng templet DNA using the primers 341F and 802R as published elsewhere (Klindworth et al. 2013). The 16S amplicons were then purified using the Agencourt AMPure XP® PCR purification system (Beckman Coulter, Krefeld, Germany), processed (indexed, purified and pooled) and sequenced in a 300-bp paired-end run using the MiSeq Reagent Kit v3.

### Taxonomic profiling of the gut microbiota

Sequencing data were processed using the DADA2 pipeline and QIIME version 2 (Callahan et al. 2016; Caporaso et al. 2010). Quality profiles of the reads were analyzed. Reads were trimmed and processed by the QIIME DADA2 plugin with the denoise-paired option and standard parameters. Rarefaction curves were determined based on the feature table, and analysis of the relative proportion of each bacterial taxon was performed after the data were rarefied at a depth of 6000 sequences per sample. Taxonomic classification was done by a Naïve Bayes classifier (sklearn) (Pedregosa et al. 2011), trained on SILVA database release 138.

Gut microbiota data of day 9 and 14 post EAN induction and/or antibiotics treatment were pooled for the further analysis.

### Tissue collection and preanalytics

Rats were anesthetized with 100 mg/kg body weight Ketamine (Inresa, Germany) and 10 mg/kg body weight Xylazine (Rompun, Germany) via i.p. injection. Under deep anesthesia, the vascular system was rinsed for 1 min with 0.1 M phosphate-buffered saline (PBS) and fixed for 20 min with approximately 500 ml 4% paraformaldehyde in 0.1 M PBS. The indicator for acceptable fixation was palpable hardening of the perfused animal’s neck muscles and liver within a few minutes. Sciatic nerves were dissected and postfixed in 4% paraformaldehyde (PFA) overnight before being cryoprotected in 30% sucrose solution at 4 °C. Subsequently, nerves were embedded in optimal cutting temperature (OCT) compound (Tissue-Tek, Japan) and 5 µm longitudinal sections were fixed on Superfrost™ Plus slides (Thermo Fisher, USA).

Duodenum tissue was dissected and postfixed in 4% PFA overnight. For paraffin-embedding, the tissue was dehydrated and cleaned following a standard protocol (Zaqout et al. 2020). Subsequently, 5 µm longitudinal sections were fixed on Superfrost™ Plus slides (Thermo Fisher, USA) and deparaffinized, rehydrated and made susceptible by heat induced antigen retrieval to a standard protocol (Zaqout et al. 2020).

Slides were made permeable with 0.2% Triton X in 1 X PBS for 15 min and blocked with normal donkey serum for 60 min at room temperature.

## Immunohistochemistry

### Sciatic nerve CD3 and Iba1 staining

For CD3 staining, slides were located in acetone for 20 min and then washed with 1 X PBS for five minutes. Subsequently, slides were blocked with 3% normal goat serum for 90 min. Thereafter, a 1:500 dilution of anti-CD3 (SP7; Abcam, Great Britain) was incubated over night at 4 °C. Afterwards, slides were washed with 2 X PBS for five minutes and a 1:500 dilution of goat-anti-rabbit AlexaFluor 568 (A78995; Thermo Fisher, USA) was used as a secondary antibody, incubating for 2 h at room temperature. Cell nuclei were stained with Hoechst 33,342 (14,533; Sigma-Aldrich, USA). Slides were ultimately washed with 2 X PBS for 10 min.

Iba1 stainings were conducted as previously described (Muke et al. 2020).

All images for this study were obtained on a BZ-9000 microscope (Keyence, Japan).

For analysis, Iba1^+^ or CD3^+^ and Hoechst nerve areas were measured and an Iba1^+^ or CD3^+^ cells-to-area ratio was calculated for three sciatic nerve sections per slide using ImageJ (Wayne Rasband, USA).

### Duodenum Zonulin, CD3/CD8 and CD3/FOXP3 staining

To assess the permeability of the duodenal tight junctions, immunoreactivity staining was used. All sections were deparaffinized and then washed thrice with distilled water for 2 min each, incubated in 0.7 g NaN3 + 80 µl H2O2 (30%) in 70 ml 1 X PBS for 20 min and washed twice with 1 X PBS for 3 min each. Slides were permeabilized with 0.2% Triton X 100 in 1 X PBS for 15 min, subsequently blocked with 5% normal goat serum (BB buffer) for 60 min and incubated overnight at 4 °C with the primary antibody (1:50 polyclonal rabbit IgG haptoglobin beta/zonulin, Bioss bs-1808R; Bioss Inc, USA). The slides were then washed twice with 1 X PBS for 5 min each time and incubated with ZytoChem Plus HRP Polymer-Anti-Rabbit (Zytomed System, Germany) as a secondary antibody for 60 min. After subsequent washing steps (twice with 1 X PBS for 5 min each), slides were incubated for 10 min in 0.1 M acetate buffer and stained with ACE at room temperature for 4 min (Sigma Aldrich, Germany). After several short washes with distilled water, all slides were coated with Kaiser's glycerol gelatin. Immunoreactivity was visualized with a Zeiss AX10 light microscope using a Hitachi HV-F202 camera. For analysis, the ratio of haptoglobin/zonulin-positive cells to area was calculated for 3 sections per intestine.

To stain CD8^+^ cytotoxic T cells, slides were stained with 1:50 anti-CD8 (Ab33786, Abcam; Great Britain) and 1:50 CD3 (ab166669, Abcam; Great Britain) and stored at 4 °C overnight. Slides were washed two times with 1 X PBS for 5 min. Subsequently, they were incubated with donkey-anti-mouse AlexaFluor 568 (A78995; Thermo Fisher, USA) and donkey-anti-rabbit AlexaFluor 488 (Ab150073; Abcam, Great Britain), diluted 1:300, for 60 min at room temperature. DAPI staining was added as indicated above. Slides were washed thrice with 1 X PBS for one minute each.

Images of CD3/CD8 positive cells were taken and counted (3 sections per intestine).

For staining of FOXP3^+^ Treg cells, a 1:50 dilution of anti-CD3 (Ab16669; Abcam, Great Britain) and of anti-FOXP3 (NB600-232; Novusbio, USA) were used. Slides were stored at 4 °C overnight and washed as shown above. Subsequently, slides were incubated with donkey-anti-rabbit AlexaFluor 488 (Ab150073; Abcam, Great Britain) and donkey-anti-goat AlexaFluor 568 (175,475; Abcam, Great Britain), diluted 1:300, for 60 min at room temperature. DAPI staining was added as described, and images of CD3/FOXP3 positive cells were evaluated (3 sections per intestine).

## Statistical analysis

Statistical analysis was performed using GraphPad Prism 9.2.0 software. Mann–Whitney U test was performed for comparison between two groups, Kruskal–Wallis or one-way ANOVA tests with Brown-Forsythe or Welchs’ post-hoc tests were performed to detect inter-group differences after testing for Gaussian distribution using Shapiro–Wilk test. Statistical analysis of the gut microbiota data and the design of the respective graphic material were performed using R for Statistical Computing® version 3.2.5 (R for Statistical Computing, Vienna, Austria) software. Shannon-Index was calculated to identify differentially abundant taxa and to compare the alpha-diversity between groups. The beta-diversity, in this case, the generalized UniFrac distances between the samples, was visualized using principal coordinate analysis (PCoA). The beta-diversity group effect was tested using a permutational multivariate analysis of variance (PERMANOVA). Regarding the analysis of the differences in the gut bacteria relative abundances, a false discovery rate-adjusted (FDR-adjusted) p-value was calculated to minimize statistical error due to multiple testing. All data are expressed as mean ± standard error of the mean (SEM) unless otherwise explained. A p-value < 0.05 was considered statistically significant.

## Results

### Antibiotics treatment alleviates EAN severity

**Fig. 1:**
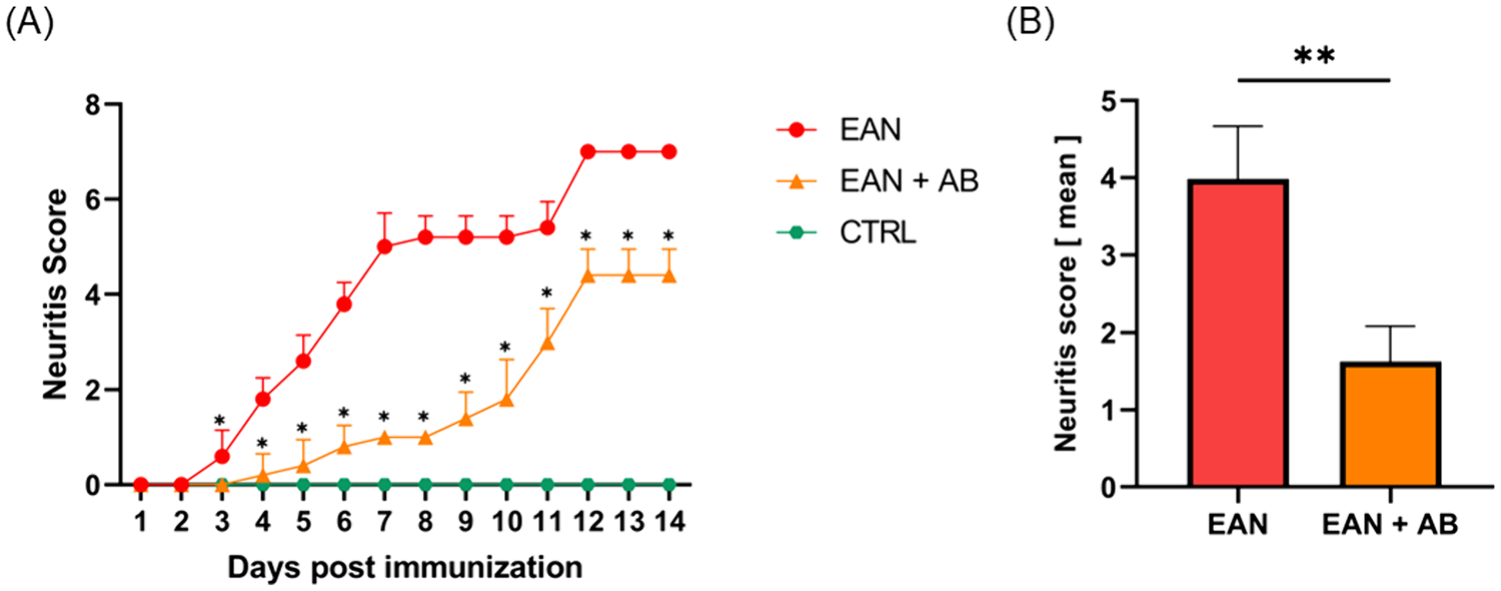
**Clinical course after EAN induction.** **A** Neuritis score in EAN rats, antibiotics-recipient EAN rats (EAN + AB), and healthy controls (CTRL). **B** EAN + AB rats showed a lower mean neuritis score (p = 0.008). *Graphs depict mean* + SEM. * p < 0.05, ** p ≤ 0.01, **** p ≤ *0.0001. Asterisks in (A) refer to significant differences between EAN and EAN* + *AB rats, significance values for comparisons with the controls are not shown*

The two EAN cohorts developed clinical symptoms at 3 days post immunization, control rats did not show any clinical signs of neuritis throughout the whole observation period (Fig. 1A and Supplemental Video 1–3). Antibiotics recipients showed significantly alleviated clinical neuritis symptoms compared to the EAN cohort without intervention, reflected by a lower mean neuritis score from three days post EAN induction onward (EAN + antibiotics [AB]: 2 ± 1 vs. EAN: 4 ± 1, p = 0.008; Fig. 1B and Supplemental Video 2).

### Antibiotics treatment reduces endoneurial T cell infiltration in EAN

**Fig. 2:**
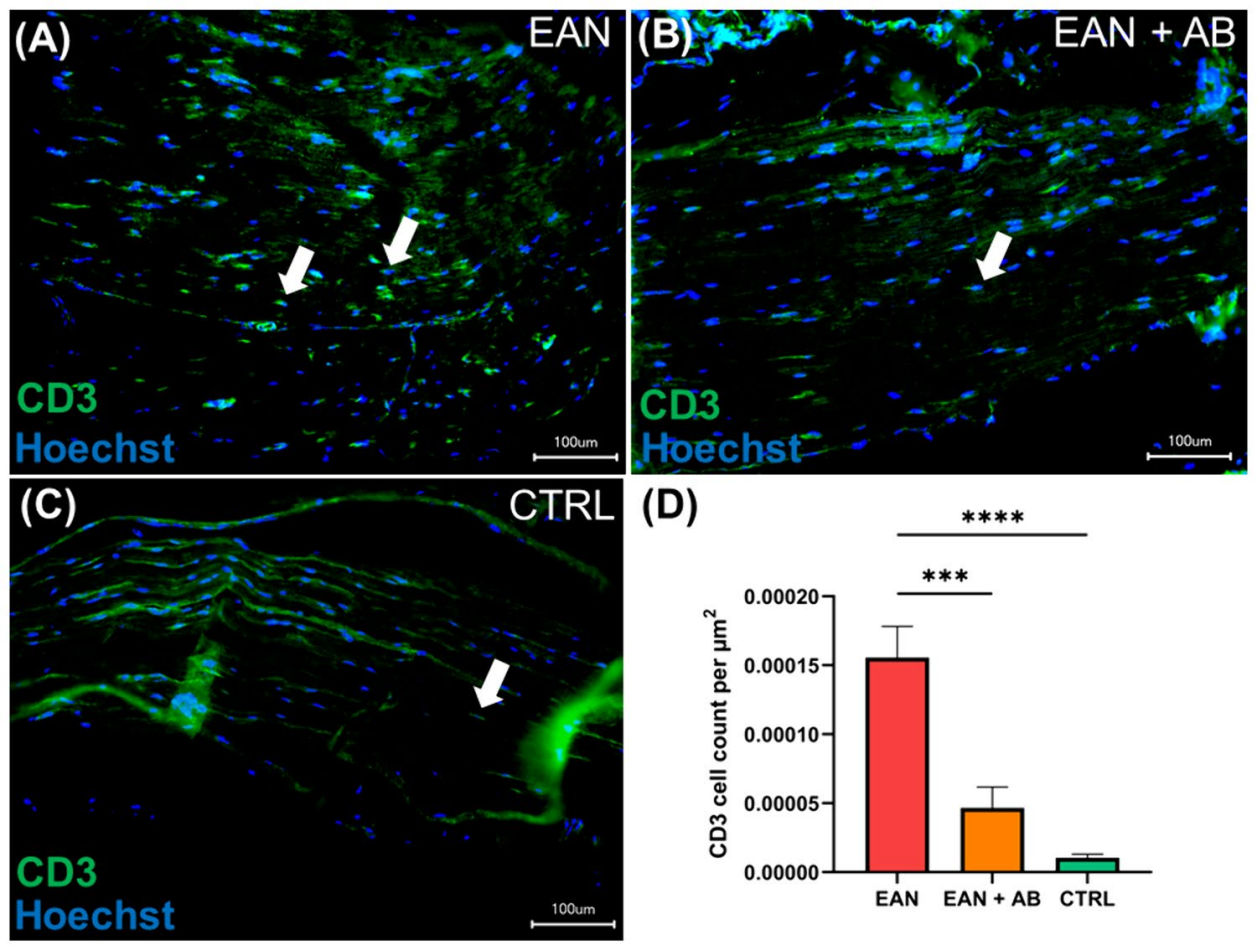
**Sciatic nerve CD3**^**+**^** T cell infiltration in EAN.** **A**-**C** Peripheral nerve tissue was stained with anti CD3 antibody (green). Cell nuclei were stained with Hoechst (blue). **D** Compared to control rats and antibiotics-recipients (EAN + AB), EAN rats without intervention showed a significant T cell increase (*p* < *0.0001)*. Scale bar = 100 µm. White arrows exemplarily indicate CD3^+^ T cells.* Graphs depict mean* + SEM. *** p ≤ 0.001, **** p ≤ *0.0001*

EAN rats that did not receive antibiotics showed significantly higher sciatic nerve CD3^+^ T cell counts than antibiotics recipients (p = 0.0008) and healthy controls (p < 0.0001; Fig. 2A-D). T cell counts did not significantly differ between antibiotics recipients and healthy controls (p = 0.13).

**Fig. 3:**
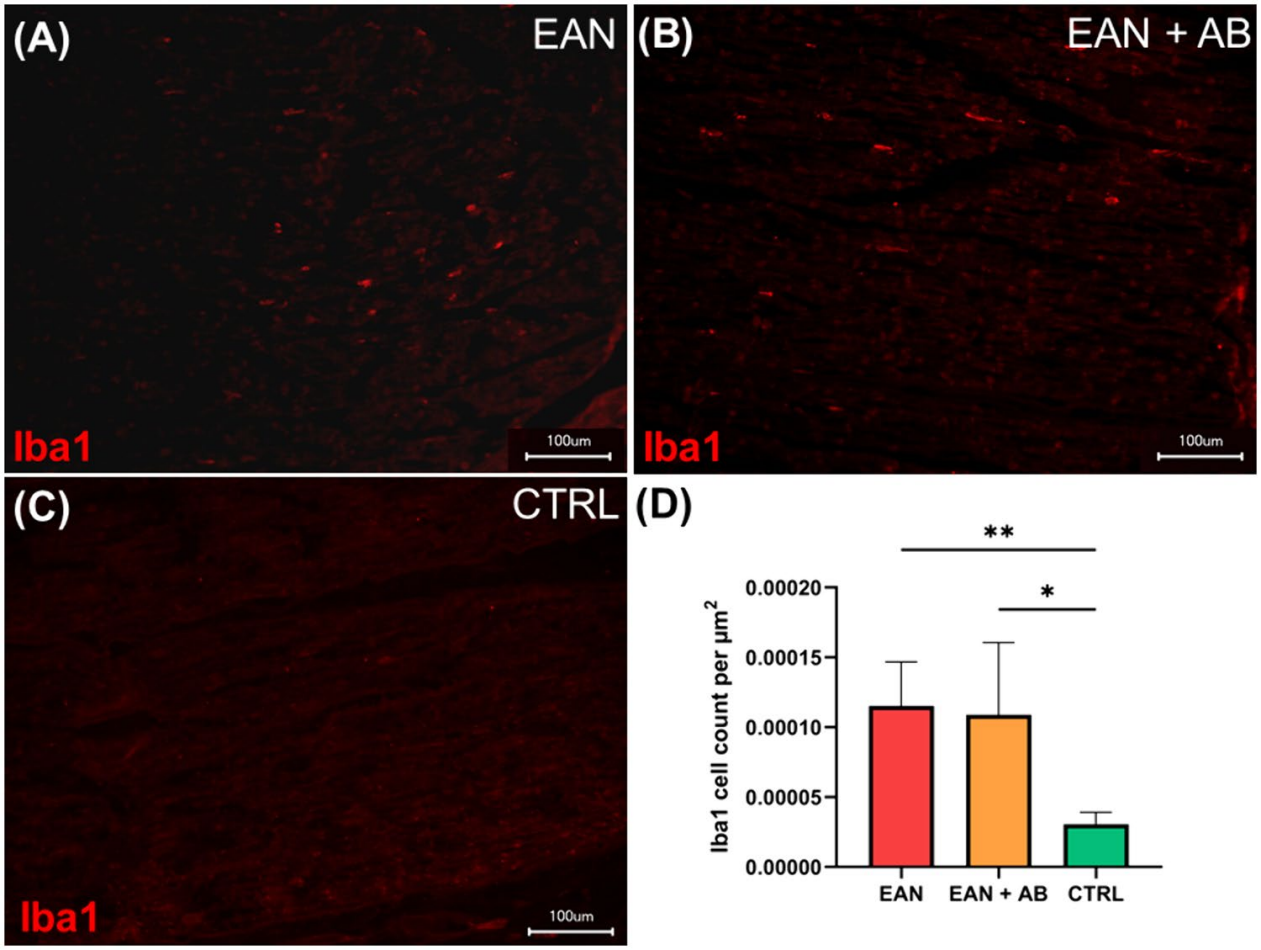
**Macrophage infiltration in EAN sciatic nerves.** **A-C** Peripheral nerve tissue was stained with anti-Iba1 antibody (red). **D** EAN rats and antibiotics-recipients EAN (EAN + AB) rats showed a significant increase of Iba1-positive (Iba1^+^) cells compared to control animals (EAN vs. CTRL p = 0.0.0066, EAN + AB vs. CTRL p = 0.0111). *Scale bar* = *100 µm. Graphs depict mean* ± SEM.* p ≤ 0.05, ** p ≤ *0.01*

EAN was associated with a significant increase of Iba1^+^ macrophages in sciatic nerves of both EAN cohorts compared to healthy controls (EAN vs. controls [CTRL]: p = 0.007, EAN + AB vs. CTRL: p = 0.01; Fig. 3A-D). Antibiotics treatment did not alter the amounts of endoneurial Iba1^+^ macrophages in EAN rats (EAN vs. EAN + AB: p = 0.96; Fig. 3A-D).

### Antibiotics treatment increases bacteria with anti-inflammatory features in EAN

**Fig. 4:**
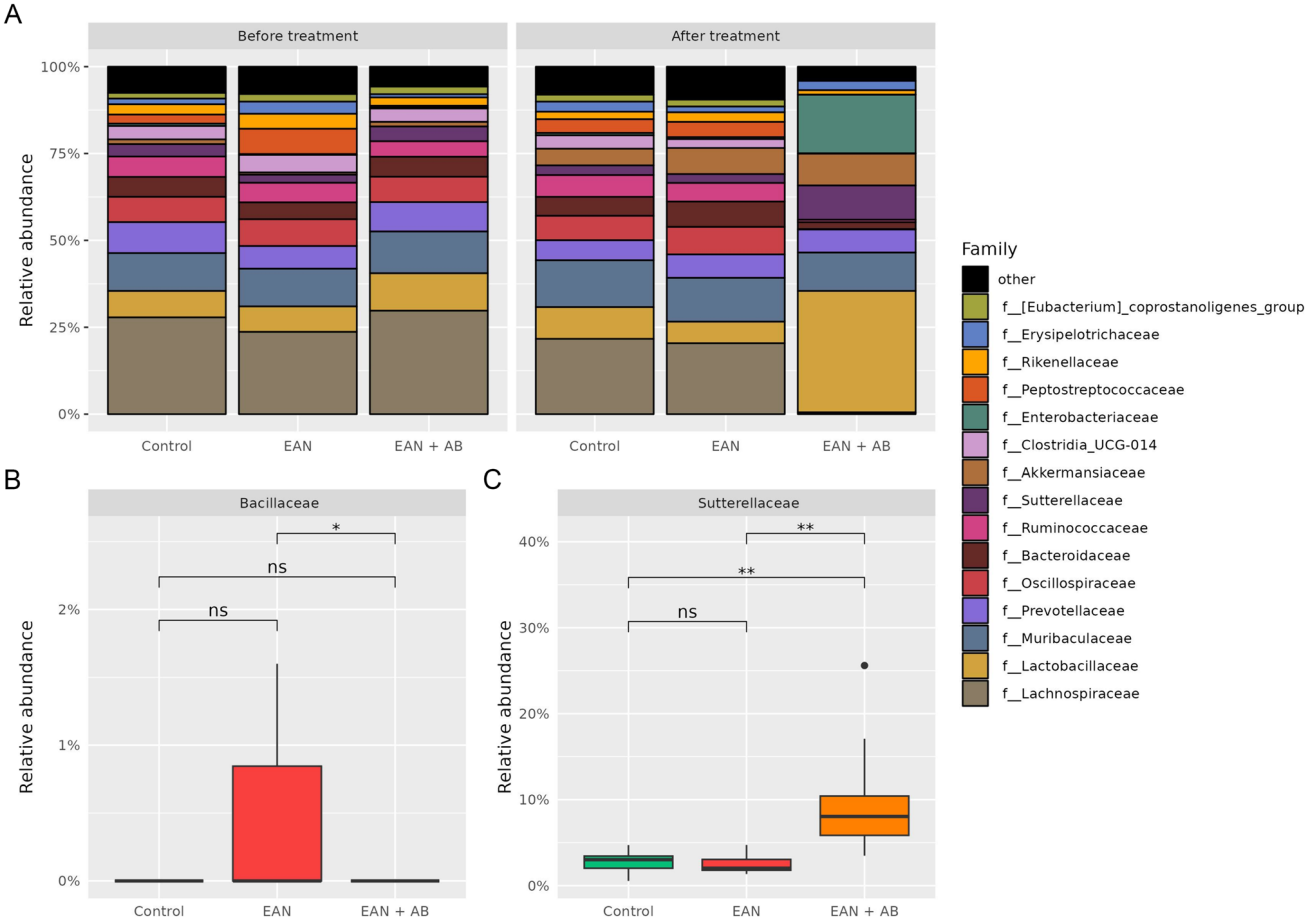
**Effect of antibiotics treatment after EAN induction on gut microbiota bacterial families. A** Taxa bar plot representing the relative abundance before and after treatment. **B**-**C** Antibiotics-treated EAN rats show a significantly lower relative abundance of *Bacillaceae* compared to the non-intervention EAN cohort, and a significantly higher abundance of *Sutterellaceae*. (Kruskal–Wallis with Dunn’s Post-hoc-Test, adjusted for multiple testing by Benjamini–Hochberg procedure)

**Fig. 5:**
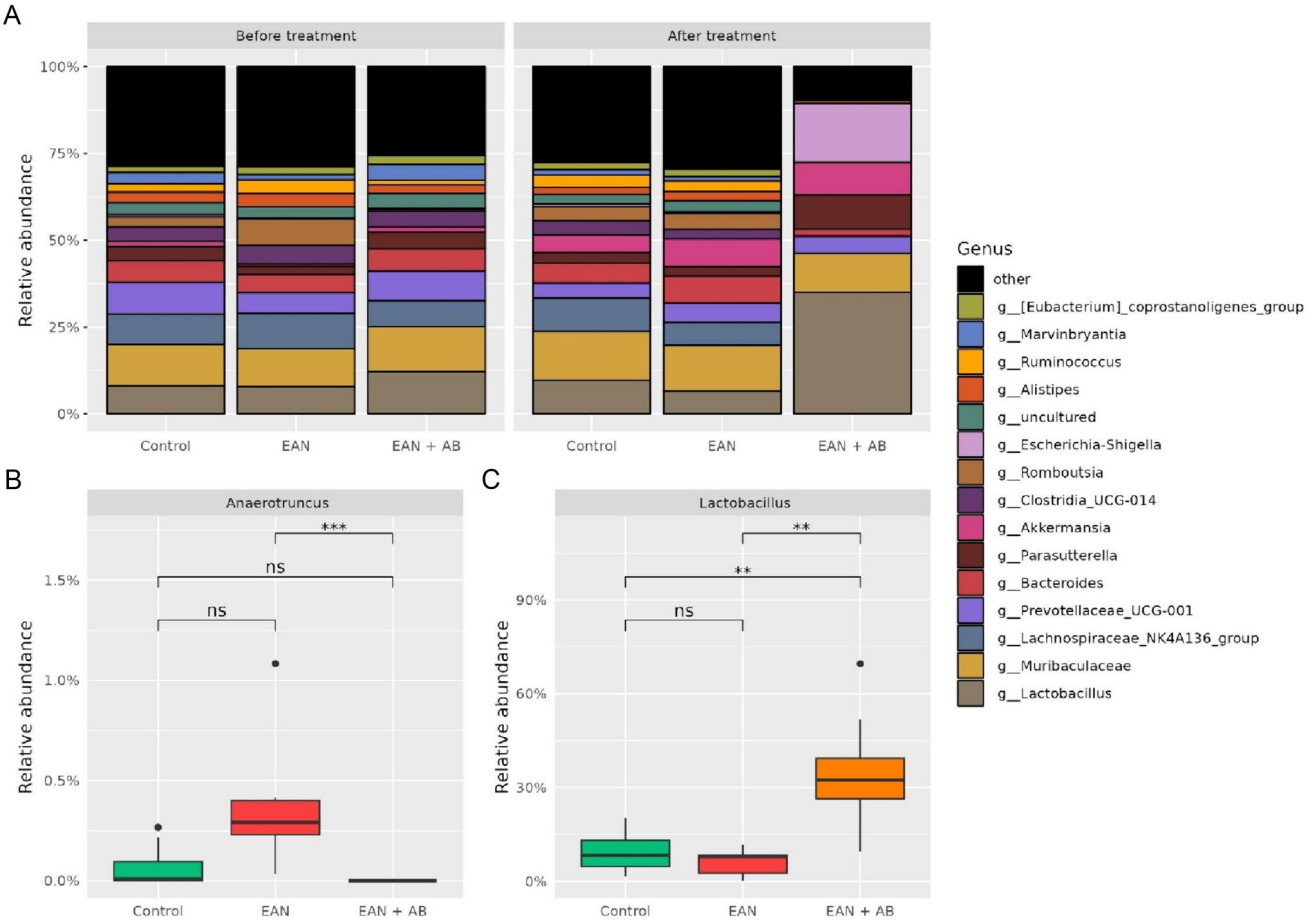
**Effect of antibiotics treatment after EAN induction on gut microbiota bacterial genus. A** Taxa bar plot representing the relative abundance before and after treatment. **B**-**C** Antibiotics-treated EAN rats show a significantly lower relative abundance of *Anaerotruncus* compared to the non-intervention EAN cohort, and i.a., a significantly higher abundance of *Lactobacillus*. (Kruskal–Wallis with Dunn’s Post-hoc-Test, adjusted for multiple testing by Benjamini–Hochberg procedure)

At baseline, prior to antibiotics treatment, gut microbiota alpha-diversities did not differ between the cohorts (p = 0.75^1^). After EAN induction, antibiotics therapy significantly reduced the alpha-diversity (Dunn’s post hoc test p = 0.001) and induced a beta-diversity (i.e. generalized UniFrac) shift (PERMANOVA: R^2^ = 0.44, F = 10, p = 0.001, Supplemental Fig. 1A-D) compared to the EAN non-intervention cohort. Gut microbiota alterations related to EAN alone did not reach statistical significance as compared to healthy controls, likely due to a relatively small cohort size. Antibiotics recipients showed higher abundances of the family *Sutterellaceae* (EAN + AB vs. EAN p = 0. 0.002, EAN + AB vs. CTRL p = 0.004) and of the genus *Lactobacillus* (EAN + AB vs. EAN p = 0.0.001, EAN + AB vs. CTRL p = 0.007) and *Parasutterella* (EAN + AB vs. EAN p = 0.002, EAN + AB vs. CTRL p = 0.004) compared to EAN rats and healthy controls (Figs. 4A + C and 5A + C and Supplemental Table 1). On the family level, *Bacillaceae*, and, on the genus level, *Anaerotruncus* and *UBA1819* were significantly reduced by antibiotics treatment in EAN rats, but not compared to healthy controls (Figs. 4B and 5B and Supplemental Table 1).

### Gut mucosal barrier dysfunction in EAN

**Fig. 6:**
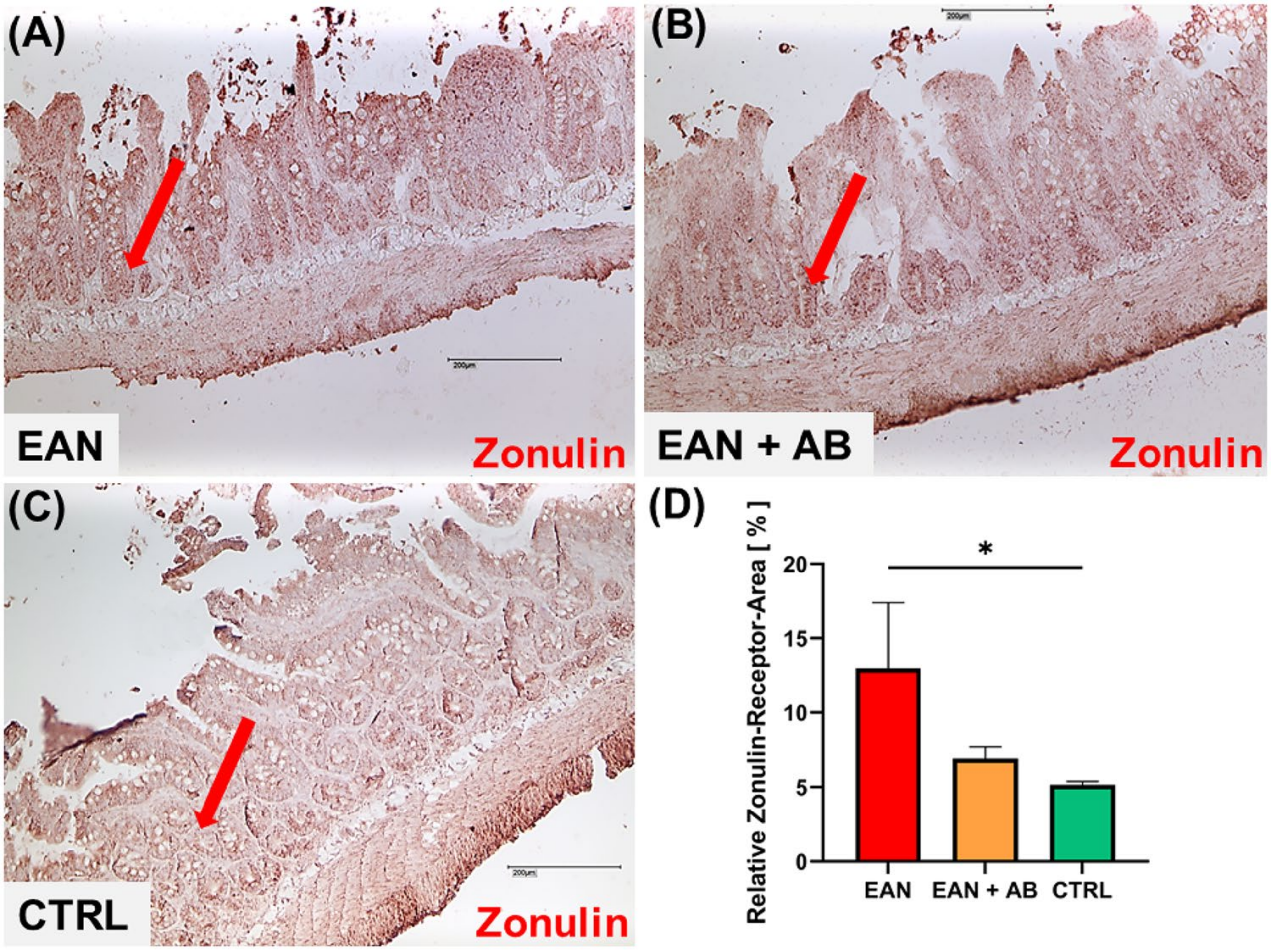
**Intestinal permeability after EAN induction**. **A**-**C** Ileum was stained with anti-zonulin antibody (red). **D** EAN animals showed a significant increase of zonulin-positive (zonulin^+^) cell areas in the mucosa compared to control animals (CTRL); p = 0.0199. Scale bar = 200 µm. Red arrows exemplarily indicate zonulin-positive areas. *Graphs depict mean* ± SEM, * p ≤ *0.05*

Zonulin levels in the duodenal mucosa were significantly higher in EAN non-antibiotics-recipients compared to healthy controls (p = 0.02; Fig. 6A-D). Antibiotics recipients showed intermediate mucosal zonulin levels that did not differ from healthy controls (p = 0.38), but also not from the non-interventional EAN cohort. (p = 0.6, Fig. 6A-D).

### Antibiotics treatment reduces ileal CD8^+^ cytotoxic T cells in EAN

**Fig. 7:**
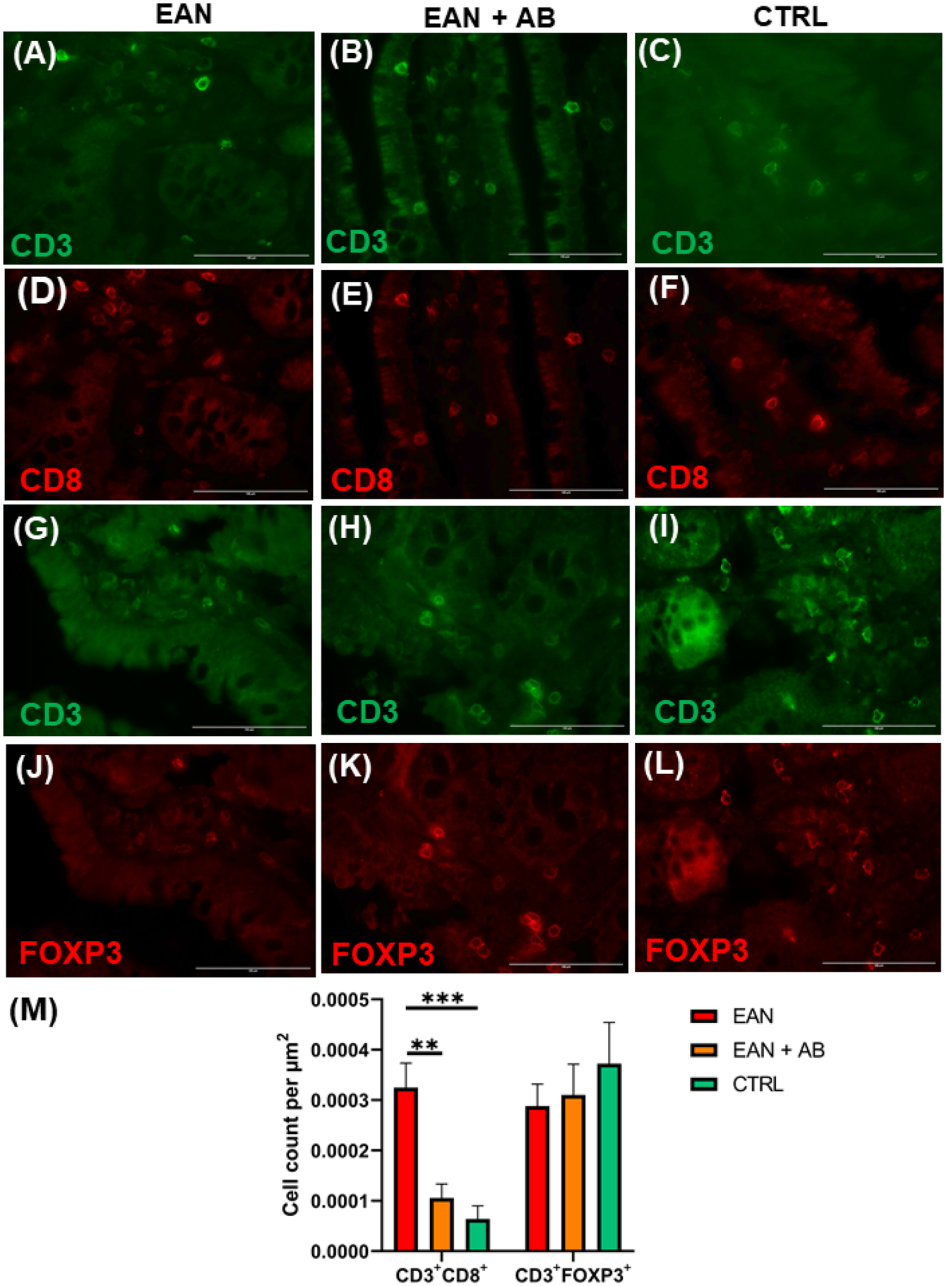
**Altered mucosal immune response after EAN induction**. The ileum was co-stained with anti-CD3/CD8 antibody (**A**-**F**, anti-CD3 green, anti CD8 red) and co-stained with anti CD3/FOXP3 (**G**-**L**, anti-CD3 green, anti-FOXP3 red). **M** EAN rats showed a significant increase of CD3/CD8-positive (CD3^+^/CD8^+^) cells compared to antibiotics-recipient EAN rats (EAN + AB) and control animals (CTRL). Scale bar= 100 µm. Graphs depict mean ± SEM. EAN vs. Controls *0.0007, EAN vs. EAN + AB ** 00027. No changes were seen in CD3/FOXP3 expression (p= 0.6400). ** p ≤ 0.01, **** p ≤ 0.0001

The amounts of ileal CD3^+^CD8^+^ cytotoxic T cells were significantly higher in the non-interventional EAN cohort compared to antibiotics recipients and healthy controls (p = 0.003/p = 0.0007; Fig. 7A-F, M). Antibiotics therapy reduced ileal cytotoxic T cells to levels of healthy controls (EAN + AB vs. CTRL: p = 0.7; Fig. 7A-F, M).

EAN and/or antibiotics treatment simultaneously did not impact intestinal CD3^+^FOXP3^+^ Treg cells (p = 0.5, Fig. 7G-L, M).

## Discussion

Our data indicate that the gut mucosa displays distinct features in EAN, which was reflected by I) increased abundances of pro-inflammatory cytotoxic T cells in gut mucosal laminae propriae and II) increased mucosal permeability as reflected by increased zonulin levels. Tendencies regarding an increase of bacteria that may be associated with pro-inflammatory mechanisms in EAN (e.g., *Bacillus* (Jezewska-Frackowiak et al. 2018), *Anaerotruncus* (Zhang et al. 2021) and *UBA1819* (Meng et al. 2023) genera), did not reach statistical significance after FDR adjustment, but did when FDR adjustment was neglected (*Bacillus*: p = 0.03, *Anaerotruncus*: 6.7 × 10^–3^, *UBA1819*: 1.7 × 10^–2^ [Kruskal–Wallis test with Dunn’s multiple comparisons test]). It is thus conceivable and likely that the unexpected and conflicting finding of lacking significant, EAN-induced gut microbiota alterations might be to some extent derived from a relatively small cohort size resulting from the 3R principles of animal experiments on one hand, and rigid false discovery rate adjustment on the other hand. Future studies, including more animals, are thus warranted to systematically characterize gut microbiota alterations in EAN, which was not the primary scope of our interventional study. On the other hand, we show that the preventive treatment of EAN rats with oral antibiotics leads to an I) intestinal and II) endoneurial immunomodulation, of which reduced gut lamina propria cytotoxic T cells, reduced sciatic nerve endoneurial T cell infiltration, and III) increased relative abundances of gut bacteria with known anti-inflammatory effects (e.g., *Sutterellaceae* and *Lactobacillus*) were key features. These modulations were associated with a significant alleviation of EAN, which is, to the authors’ knowledge, the first demonstration of beneficial effects of an antibiotics-induced intestinal immunomodulation on the course of EAN.

As mentioned, this immunomodulation was reflected by lower lamina propria cytotoxic T cells in EAN rats that received antibiotics treatment for two weeks since EAN induction, and alike by an alleviated endoneurial T cell infiltration in these animals. Cytotoxic T cells play a crucial role in inflammatory processes, as they secrete pro-inflammatory cytokines such as IFN-ɣ after activation. In EAN, CD8^+^ cytotoxic T cells (alongside autoreactive T-helper cells) are main executors of the autoinflammatory response to peripheral nerves (Zhu et al. 2002). It is assumed that T cells recognize self-antigens and subsequently initiate autoinflammation in immune neuropathies (Mausberg et al. 2018), and their pathogenic role has been underlined in several adoptive transfer studies, where transferred neuritogenic T cells of EAN animals induced EAN in recipients (Linington et al. 1986). A previous study found increased cytotoxic T cell and autoreactive T-helper cell subsets in the blood, cerebrospinal fluid (CSF), and nerve tissue of patients with acute inflammatory demyelinating polyneuropathy (AIDP), the demyelinating GBS variant, supporting that cytotoxic T cells play a central role in GBS (Súkeníková et al. 2024).

Our finding of decreased lamina propria cytotoxic T cell abundances after antibiotics treatment in association with alleviated clinical EAN severity further supports the hypothesis that cytotoxic T cells play a key role in EAN.

T cells in the intestinal lamina propria have a two-sided function: on one hand, they provide protection against dietary and enteric microbial antigens as well as pathogens, on the other hand, they maintain tissue-specific and systemic immunological homeostasis as they modulate exoantigen tolerance (Levine and Fiocchi 2001).

A scrupulous homeostasis of gut permeability is essential to maintain a physiological interplay between exoantigens and lamina propria T cells, and its disruption due to increased mucosal leakiness is associated with changes in the gut microbiota (i.e. reduced abundances of short-chain fatty acids producing bacteria (Ordoñez-Rodriguez et al. 2023)) in a variety of autoimmune diseases (Kaur et al. 2022). Suitably, patients with autoimmune diseases often show a leaky gut mucosa (Kinashi and Hase 2021), which is in line with our findings in EAN, where an increased mucosal zonulin expression was observed compared to healthy rats. Zonulin, as a component of intercellular tight junctions, regulates the molecular trafficking between the intestinal lumen and submucosa, leading to either immune response or tolerance, and positively correlates with gut permeability (Fasano 2020). Our data therefore indicate an increased gut permeability, which is very likely to result in an increased interaction of microbial components and mucosal T cells, leading to our observation of increased mucosal pro-inflammatory cytotoxic T cell counts in EAN. As these findings are in line with a more mechanistic study on mucosal CD8^+^ T cells, that demonstrated an important pathogenic role in MS (Abrahamsson et al. 2013), we are confident that our findings in the EAN gut mucosa are associated with a triggered systemic immune response, promoting inflammatory T cell infiltration into peripheral nerves. However, further studies are warranted to add more mechanistic knowledge on the interplay between gut mucosal cytotoxic T cells and endoneurial T cell infiltration.

Although our study did not directly investigate the interplay between gut mucosal and endoneurial T cell subsets in EAN, the fact that a significant alleviation of EAN symptom severity alongside reduced endoneurial T cell infiltration was observed after oral antibiotics treatment, makes such a gut mucosa – nerve axis likely to be involved in the pathogenesis of EAN. This is further supported by the fact that antibiotics specifically target the proliferation of bacteria, for which the gut is the main reservoir, and not eukaryotic cells, what makes direct endoneurial-antibiotics effects very unlikely to explain our results (Donaldson et al. 2015; Patangia et al. 2022).

The fact that antibiotics-induced intestinal immunomodulation only affected endoneurial T cells and not Iba1^+^ macrophage counts appears conflicting, as macrophages are key effectors of T cell-mediated nerve damage in autoimmune neuropathies, phagocyting myelin and axonal proteins (Griffin et al. 1990). However, T cells play the most important role in the recruitment of macrophages to peripheral nerves in EAN (Mausberg et al. 2011; Xu et al. 2019), and the fact that our study did not further investigate endoneurial macrophage polarization could mask beneficial effects of the antibiotics treatment on macrophage function, as observed in other studies that showed an upregulation of anti-inflammatory M2 macrophages in EAN nerves after therapeutic interventions (Mausberg et al. 2011, 2018; Xu et al. 2019). Future studies on beneficial effects of antibiotics in EAN should therefore also elucidate macrophage polarization, and consider to encompass a longer observational period, as our study terminated at day 14 post EAN induction, which although reflects the time point of peak EAN symptom severity and inflammatory activity (Xu et al. 2019).

Many studies reveal preclinical and clinical evidence that gut microbiota play a role in immune homeostasis and that imbalance in the gut microbiota can contribute to the modulation of neuroinflammation (Kaur et al. 2022; Ordoñez-Rodriguez et al. 2023). Germ-free mice did not develop EAE and EAE mice that received microbiota of donors housed in specific pathogen-free conditions, experienced a clinical amelioration (Berer et al. 2011). Based on this observation, and the observation by Yokote and colleagues, that antibiotics treatment (kanamycin, vancomycin and kolistin) significantly suppressed the development of EAE by altering the gut microbiota, associated with less neuroinflammation characterized by a downregulation of pro-inflammatory cytokines and less pro-inflammatory mesenteric Th17 cells (Yokote et al. 2008), our results align with pointing out that eradication/modulation of gut bacteria has beneficial effects in demyelinating autoimmune diseases.

Furthermore, previous studies showed distinct features in the composition of the gut microbiota in autoimmune diseases of the PNS and central nervous system (CNS). For example, MS patients exhibit increased abundances of some bacterial genera, such as *Akkermansia*, *Blautia* and *Methanobrevibacter* (Correale et al. 2022), while patients with CIDP reveal increased abundances of Firmicutes such as *Blautia*, *Ruminococcus torques*, and others (Svačina et al. 2023).

In our study, antibiotic treatment resulted in increased *Sutterellaceae* on a family level and in increased *Lactobacillus* and *Parasutterella* on a genus level.

It is well known that *Lactobacillus species* have a beneficial role in modulating human health and they are widely used as prebiotics. They are short chain fatty acid (SCFA)-producers, which results in upregulated mucin secretion and decreased pro-inflammatory cytokine secretion and therefore, enhancing the intestinal barrier shielding and reducing inflammation (Dempsey and Corr 2022). This could have a beneficial impact on the mucosal CD8^+^ T cells, leading to a reduced inflammatory priming. This would also align with a study that revealed anti-inflammatory effects of *Lactobacillus casei* resulting in less CD8^+^ T cell-mediated skin inflammation (Chapat et al. 2004).

Compared to the control group, our EAN rats did not show a decrease of *Lactobacillus*. The upregulation of *Lactobacillus* after antibiotic treatment could be due to the immunomodulatory effects of antibiotics itself. It is well known that antimicrobial substances not only combat the respective bacteria but also contribute to reconstitute intestinal immune homeostasis by having an anti-inflammatory effect (Pradhan et al. 2016). This data is in line with the work of Meng and colleagues, in which a substitution of *Lactobacillus paracasei* L9 in EAN rats resulted in a reduced disease progression by regulating gut microbiota and arginine metabolism (Meng et al. 2023).

*Sutterellaceae* and *Parasutterella* have benefical effects on host health (Giri and Mangalam 2019; Ju et al. 2019; Xu et al. 2021). For example, *Sutterellaceae* were found to be decreased in EAE mice and MS patients and were increased after a disease-modifying therapy such as interferon-beta (Giri and Mangalam 2019). Previous studies observed a negative correlation between *Parasutterella* abundance and high-fat-diet-induced metabolic phenotypes including hypothalamic inflammation (Kreutzer et al. 2017; Zhang et al. 2012). In our EAN rats, bacteria of the family *Sutterellaceae*, especially the genus *Parasutterella*, were increased after antibiotic treatment, suggesting an immunomodulatory effect protecting from autoimmunity and inflammation.

There are several limitations to this study. Firstly, we did not perform nerve conduction studies or axon counting on the peripheral nerves. Although the neuritis score is a well-established measure for EAN severity, future studies should also encompass the mentioned additional measures to further confirm our findings. Furthermore, our study delineated short-term effects of an antibiotics-induced intestinal immunomodulation, providing data hinting to a gut mucosa – peripheral nerve immune axis, but more mechanistic analyses (e.g., via leukocyte trafficking or metabolomic studies) are warranted to identify the exact pathways that contribute to the observed immunomodulation in EAN, and should also provide a longer observation period to assess possible effects on nerve regeneration post EAN.

Nevertheless, this study is the first to provide valid data on a gut mucosa – peripheral nerve immune axis in EAN, pointing out that antibiotics-induced intestinal immunomodulation might be a possible approach in immune neuropathies.

## Conclusion

In summary, our study demonstrates that mucosal immunity plays a role in EAN, and that an antibiotics-induced intestinal immunomodulation exerts beneficial systemic effects by alleviating EAN severity, which might offer a translational value to patients with immune-mediated neuropathies. However, this has to be confirmed by future translational studies.

## Supplementary Information

Below is the link to the electronic supplementary material.

Supplementary Figure 1: **Comparison of the gut microbiota before and after EAN induction. **(**A-B**) EAN induction did not significantly alter the alpha diversity of the gut microbiota (Kruskal-Wallis-Test p = 0.68, Post-hoc-Test: Dunn-Test). (**A-D**) After the treatment, uniquely, the antibiotics recipients EAN rats (EAN+AB) showed a significantly reduced alpha-diversity (Kruskal-Wallis-Test p < 0.001, Post-hoc-Test: Dunn-Test) and a shift in beta-diversity (PERMANONA r^2^= 0.436, p = 0.001) (JPG 397 KB)

Supplementary Table 1: **Overview of bacterial family and genus relative abundances after antibiotic treatment in EAN. **Significant increases and decreases in relative abundance values that persisted after FDR-adjustment (Benjamini-Hochberg procedure) are considered to be increased or decreased in EAN or antibotics recipient EAN rats (EAN+AB) compared to each other and to controls. An FDR-adjusted p-value < 0.05 is considered to be statistically significant. Timepoints refer to pre and post EAN induction. (XLSX 11 KB)

Supplementary Video 1: **Clinical symptoms after EAN induction.** EAN rats developed clinical symptoms at day 3 post immunization (MP4 38816 KB)

Supplementary Video 2: **Antibiotics treatment alleviates EAN severity. **Antibiotic recipient EAN rats showed significantly alleviated clinical neuritis symptoms compared to the EAN cohort without intervention (MP4 2215 KB)

Supplementary Video 3: **Antibiotics treatment alleviates EAN severity. **The control group did not show any clinical neuritis symptoms (MP4 35176 KB)

## Data Availability

The data that support the findings of this study are available on reasonable request from the corresponding author.

## Footnotes

1 1 Hereafter, all p-values in the manuscript are FDR-adjusted if not differently described.

## Glossary

AB: antibiotics
AIDP: acute inflammatory demyelinating polyneuropathy
CIDP: chronic inflammatory demyelinating polyneuropathy
CNS: central nervous system
CSF: cerebrospinal fluid
CTRL: control
DNA: desoxyribonucleic acid
EAE: experimental autoimmune encephalomyelitis
EAN: experimental autoimmune neuritis
FDR: false discovery rate
GBS: Guillain-Barré syndrome
ml: milliliter
MS: Multiple Sclerosis
OCT: optimal cutting temperature
PBS: phosphate-buffered saline
PFA: paraformaldehyde
PNS: peripheral nervous system
rRNA: ribosomal ribonucleic acid
µl: microliter
