# Supplementary figures and images for: Antibiotics-Induced Intestinal Immunomodulation Attenuates Experimental Autoimmune Neuritis (EAN)
Source: J Neuroimmune Pharmacol. 2024 May 31;19(1):26. doi: 10.1007/s11481-024-10119-9 (PMC11143056; doi:10.1007/s11481-024-10119-9)

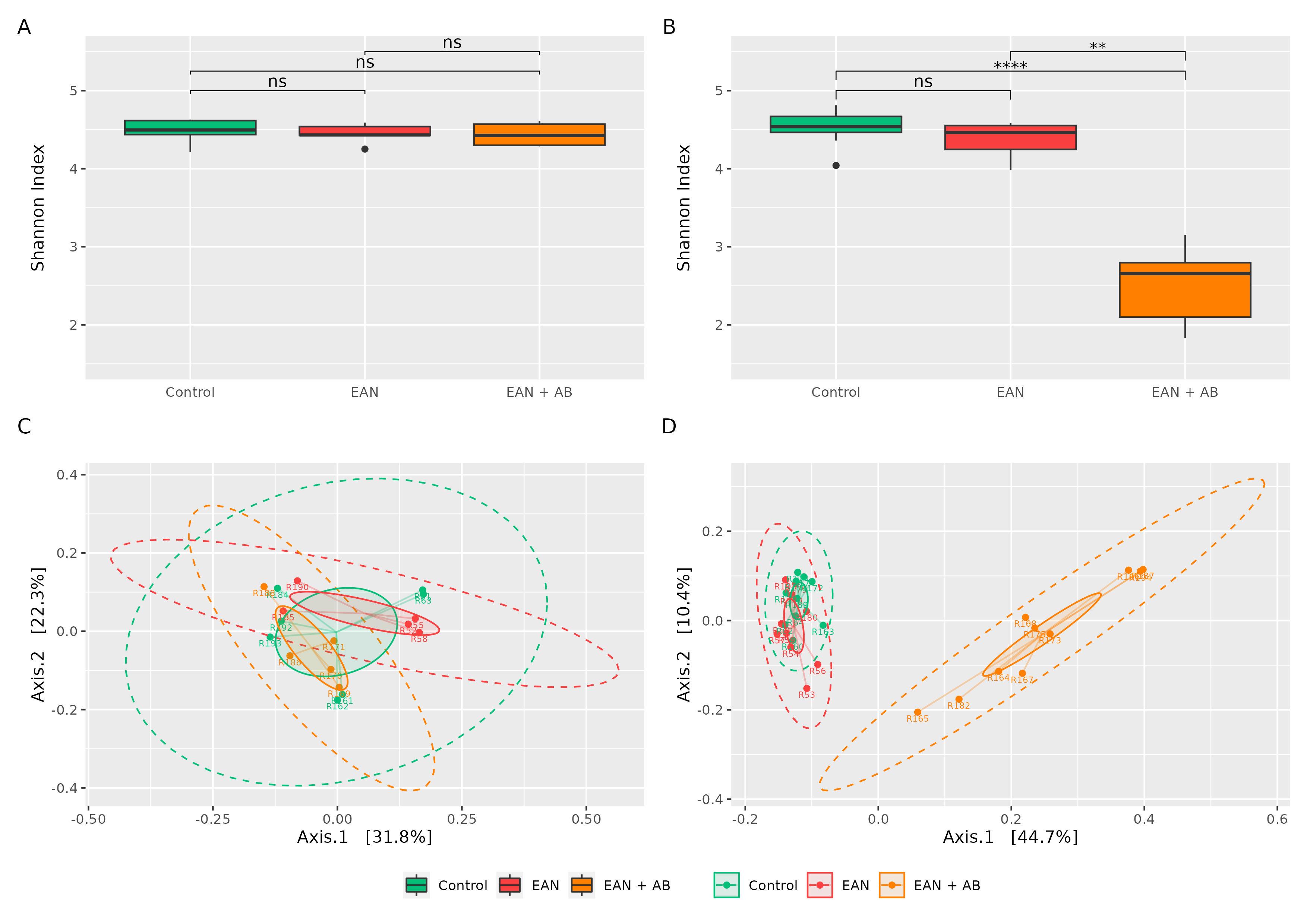

Supplement: Supplementary file 1 — Supplementary Figure 1: Comparison of the gut microbiota before and after EAN induction. (A-B) EAN induction did not significantly alter the alpha diversity of the gut microbiota (Kruskal-Wallis-Test p = 0.68, Post-hoc-Test: Dunn-Test). (A-D) After the treatment, uniquely, the antibiotics recipients EAN rats (EAN+AB) showed a significantly reduced alpha-diversity (Kruskal-Wallis-Test p < 0.001, Post-hoc-Test: Dunn-Test) and a shift in beta-diversity (PERMANONA r2= 0.436, p = 0.001) (JPG 397 KB) [file 11481_2024_10119_MOESM1_ESM.jpg]
